# Oxidative stress-triggered Wnt signaling perturbation characterizes the tipping point of lung adeno-to-squamous transdifferentiation

**DOI:** 10.1038/s41392-022-01227-0

**Published:** 2023-01-11

**Authors:** Zhaoyuan Fang, Xiangkun Han, Yueqing Chen, Xinyuan Tong, Yun Xue, Shun Yao, Shijie Tang, Yunjian Pan, Yihua Sun, Xue Wang, Yujuan Jin, Haiquan Chen, Liang Hu, Lijian Hui, Lin Li, Luonan Chen, Hongbin Ji

**Affiliations:** 1 State Key Laboratory of Cell Biology, Shanghai Institute of Biochemistry and Cell Biology, CAS Center for Excellence in Molecular Cell Science, Chinese Academy of Sciences, Shanghai, 200031 China; 2 University of Chinese Academy of Sciences, Beijing, 100049 China; 3 Zhejiang University-University of Edinburgh Institute, Zhejiang University School of Medicine, Haining, 314400 China; 4 Department of Thoracic Surgery, Fudan University Shanghai Cancer Center, Shanghai, China; 5 Department of Oncology, Shanghai Medical College, Fudan University, Shanghai, China; 6 School of Life Science and Technology, ShanghaiTech University, Shanghai, 200120 China; 7 State Key Laboratory of Molecular Biology, Shanghai Institute of Biochemistry and Cell Biology, CAS Center for Excellence in Molecular Cell Science, Chinese Academy of Sciences, Shanghai, 200031 China; 8 Key Laboratory of Systems Health Science of Zhejiang Province, School of Life Science, Hangzhou Institute for Advanced Study, University of Chinese Academy of Sciences, Chinese Academy of Sciences, Hangzhou, 310024 China

**Keywords:** Cancer models, Lung cancer

## Abstract

*Lkb1* deficiency confers the *Kras*-mutant lung cancer with strong plasticity and the potential for adeno-to-squamous transdifferentiation (AST). However, it remains largely unknown how *Lkb1* deficiency dynamically regulates AST. Using the classical AST mouse model (*Kras* ^*LSL-G12D/+*^*;Lkb1*^*flox/flox*^, *KL*), we here comprehensively analyze the temporal transcriptomic dynamics of lung tumors at different stages by dynamic network biomarker (DNB) and identify the tipping point at which the Wnt signaling is abruptly suppressed by the excessive accumulation of reactive oxygen species (ROS) through its downstream effector FOXO3A. Bidirectional genetic perturbation of the Wnt pathway using two different *Ctnnb1* conditional knockout mouse strains confirms its essential role in the negative regulation of AST. Importantly, pharmacological activation of the Wnt pathway before but not after the tipping point inhibits squamous transdifferentiation, highlighting the irreversibility of AST after crossing the tipping point. Through comparative transcriptomic analyses of mouse and human tumors, we find that the lineage-specific transcription factors (TFs) of adenocarcinoma and squamous cell carcinoma form a “Yin-Yang” counteracting network. Interestingly, inactivation of the Wnt pathway preferentially suppresses the adenomatous lineage TF network and thus disrupts the “Yin-Yang” homeostasis to lean towards the squamous lineage, whereas ectopic expression of NKX2-1, an adenomatous lineage TF, significantly dampens such phenotypic transition accelerated by the Wnt pathway inactivation. The negative correlation between the Wnt pathway and AST is further observed in a large cohort of human lung adenosquamous carcinoma. Collectively, our study identifies the tipping point of AST and highlights an essential role of the ROS-Wnt axis in dynamically orchestrating the homeostasis between adeno- and squamous-specific TF networks at the AST tipping point.

## Introduction

Lung cancer, the devastating disease with high mortality, is notorious for its extremely high heterogeneity and strong plasticity. Two main histological types of non-small-cell lung carcinoma (NSCLC, ~86% of lung cancer), adenocarcinoma (ADC) and squamous cell carcinoma (SCC), are characterized by their respective lineage transcription factors (TFs): *NKX2-1* for ADC, and *TP63* for SCC.^1–7^ Perturbation of those lineage-specific TFs may result in the destabilization and loss of the corresponding cellular identity.^6,8,9^ Interestingly, the existence of mixed adenosquamous (AdSCC) histology in clinic^10,11^ and the largely shared genetic alterations between these two histological portions^12–14^ indicate potential phenotypic transition, which is known as the adeno-to-squamous transdifferentiation (AST).^15–17^ Squamous transformation is recently observed in relapsed *EGFR*-mutant lung ADC patients after initially successful tyrosine kinase inhibitor (TKI) treatments.^16,18^ Moreover, a recent study shows that squamous transformation becomes prevalent in *EGFR*-mutant lung ADC patients relapsed from the treatment of Osimertinib, the third-generation TKI.^18^ Similar phenomena are also observed in 2/9 of lung ADC patients after the acquisition of KRAS^G12C^ inhibitor resistance.^19^ These studies have highlighted an important contribution of AST to the acquisition of molecular targeted therapy resistance in lung cancer clinics.

*LKB1* (also named as serine-threonine kinase 11, STK11) is mutated in ~17% human lung ADC^20^ whereas its mutation rate is enriched in lung AdSCC, averaging at 39.66% (ranging from 22 to 66% in multiple studies).^12,20–23^ A recent study has analyzed *LKB1* mutations in relapsed patients with potential adeno-to-squamous transdifferentiation, which shows a relatively low rate at 14.3% (1 out of 7).^20^ We reason that such low *LKB1* mutation rate might be ascribed to the mutual exclusivity between *LKB1* and *EGFR* mutations^24–26^ since most patients harbor *EGFR* genetic alterations, e.g., 4/7 with *EGFR* mutations and 1/7 with *EGFR* amplification.^20^ Excluding these *EGFR*-altered patients, the *LKB1* mutation rate ranges from 33 to 50%, similar to previous reports.^12,20–23^ These clinical observations collectively indicate a potential role of *LKB1* mutations in driving AST. Consistently, we have previously found that *Lkb1* inactivation is able to drive lung cancer AST in the *Kras*^*G12D*^-based genetically engineered mouse models (GEMM).^23,27–29^ These pathologically transitioned mouse tumors also display therapeutic resistance to multiple inhibitors initially effective in lung ADC,^29^ similar to the clinical observations.

In the *KL* mouse model,^30^ ADC typically arises at 6 weeks post nasal inhalation of Adeno-Cre (Ad-Cre) and subsequently transdifferentiates into SCC at 8 weeks.^28,29^ *LKB1*-deficiency can trigger strong metabolic imbalance and excessive accumulation of reactive oxygen species (ROS).^29^ Such uncontrolled oxidative stress subsequently promotes the transdifferentiation from adenomatous pathology to squamous pathology.^29^ However, the underlying mechanisms remain largely unknown. Moreover, the transcriptomic and molecular dynamics of the *Lkb1*-loss-driven AST process, which should be crucial to uncover such mechanisms, have not yet been characterized.

From the system dynamics perspective, AST is among a category of nonlinear processes known as critical transition, which has been widely found in physiological systems, ecological systems, climate systems, and social systems.^31–35^ In general, such transition involves a drastic switch in the system state as it approaches the critical threshold referred to as the tipping point (TP).^34^ Before passing through the tipping point, the system state gradually changes but still holds its original state. As it gets close to the tipping point, a slight incremental change or even some stochastic noise could cause a catastrophic shift, which eventually brings the state to an alternative state and makes it difficult to return to the original state.^36,37^ In other words, the same perturbation on the system state before or after the tipping point might have different effects. It would therefore be important to detect the tipping point,^36^ which not only provides early-warning signals of disease progression but also identifies potentially important driving factors. Based on the non-linear dynamical theory, we have previously developed a network-based indicator of tipping point, i.e., dynamic network biomarker (DNB), which could identify the dominant genes controlling the TP based on differential covariance rather than differential gene expression commonly used in traditional bioinformatic analyses.^36,38–41^ The covariance includes both the correlations and deviations among genes. Notably, DNB has been recently successfully applied to multiple biological transitions including virus infection, disease onset, and cancer metastasis.^36,38–40^

Taking advantage of the *KL* mouse model for AST study, we here systematically characterize the temporal transcriptomic dynamics of lung tumors, delineate the non-linear dynamical process, and further identify the oxidative stress-triggered Wnt inactivation as the tipping-point regulator during the AST process. We propose a model for the AST process involving the Wnt inactivation-mediated disruption of the ADC- and SCC-lineage-specific transcriptional factors counteracting “Yin-Yang” network.

## Results

### Temporal transcriptomic characterization of AST

**Fig. 1:**
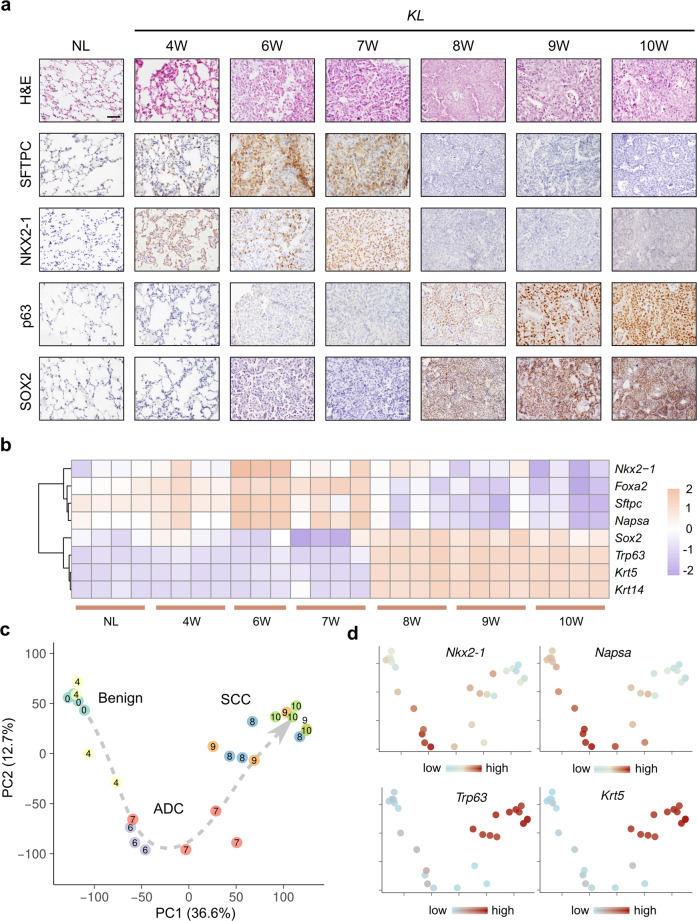
Temporal transcriptomic characterization of AST in *KL* mouse model. **a** Representative H&E staining and immunohistochemistry (IHC) photos of *KL* mouse lungs at a series of time points after Ad-Cre nasal inhalation, ranging from 4 weeks (4 W) to 10 weeks (10 W). 4W-7W were ADC and 8W-10W were SCC. Normal lung (NL) were used as control. Scale bar: 50 µm. **b** RNA-seq analyses of *KL* lung lesions at a series of time points. Relative expression of canonical ADC and SCC marker genes were depicted. **c** Principal component analyses (PCA) of RNA-seq data revealing the trajectory of phenotypic transitions from benign tissue to ADC to SCC. Each node represented one sample, with the number indicating the time points (weeks) of sampling. 0 indicated NL. **d** Transcriptional dynamics of ADC markers (Nkx2-1, Napsa) and SCC markers (Trp63, Sox2) on the same PCA embedding as **c**. Node colors reflected the relative expression of each marker gene

To uncover the temporal transcriptomic dynamics of lung tumors during the AST process, we took advantage of the *KL* mouse model, the well-established AST model.^23,27,28^ Consistent with the previous study,^28^ we found that lung ADC was evident at 6 weeks post Ad-Cre administration, whereas SCC began to emerge at 8 weeks. To monitor the temporal transcriptomic dynamics during the transdifferentiation process, we took a serial-sampling strategy post Ad-Cre administration for RNA-seq analyses. The mouse specimens were respectively atypical adenomatous hyperplasia (AAH) at 4 weeks (4 W), ADC at 6 and 7 weeks (6 W and 7 W), SCC at 8, 9, and 10 weeks (8 W, 9 W, 10 W) post Ad-Cre administration, which were validated by histopathological analyses and immunostaining of ADC markers including SFTPC and NKX2-1, and SCC markers including p63 and SOX2 (Fig. 1a). Normal lung (NL) tissues were also included as the control. The transcriptomic data showed a pattern of marker gene expression consistent with the histopathological analyses (Fig. 1b). After the dimension reduction with principal component analysis (PCA) and visualization of the samples on the first two principal components, we observed a clear trajectory of state transition, following three major phases consecutively: benign state (NL or AAH), to ADC, and eventually to SCC (Fig. 1c). Compared to the ADC at 6 W, the 7 W ADC showed a higher variability at transcriptomic level (Fig. 1c) as well as individual biomarker gene expression level, e.g., the ADC lineage markers *Nkx2-1* and *Napsa* (Fig. 1b, d). This indicates that the 7 W ADC is at a relatively unstable state. This promoted us to treat the AST event as a non-linear process mediated by critical transition, using the theories and methods developed for dynamical systems.

### Dynamic network biomarker analyses reveal the tipping point of AST

**Fig. 2:**
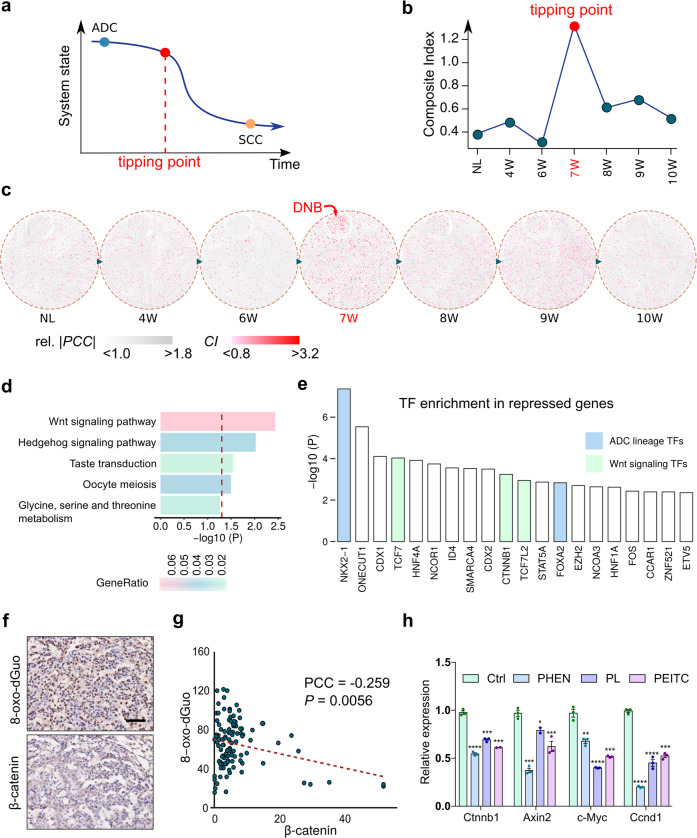
Dynamic network biomarker analyses reveal the tipping point of AST and its regulatory signaling. **a** Schematic diagram of a theoretical model for system state transition during AST. As lung tumors phenotypically switched from adenocarcinoma (ADC) to squamous carcinoma (SCC), three general phases could be observed: ADC phase, critical phase, SCC phase. The tumor state first gradually changes during the ADC phase, then reaches the tipping point (TP) before the drastic changes, and finally reaches a second slow-changing SCC state. **b** A composite index (CI) for quantifying the tipping point of system state. The peak of CI at 7 W indicates the tipping point. **c**. Network dynamics along the sampling time points during the AST process. A key module, called the dynamic network biomarker (DNB), is detected as the critical state at the 7 W (tipping point). Edges were colored by normalized absolute Pearson’s correlation coefficient (PCC). Nodes were colored by individual CI scores. **d** KEGG pathway enrichment for the DNB genes. Top 5 most significant pathways were shown. **e** TF enrichment in the upstream of genes repressed across the tipping point. Top 20 TFs are ranked with −log10(*P* value) and the ADC lineage-specific TFs and Wnt signaling related TFs were highlighted. **f** Representative photos for immunohistochemical staining of 8-oxo-dGuo and β-catenin in *KL* mouse lung tumors at 7 W post Ad-Cre treatment. Scale bar: 50 µm. **g** Correlation of immunohistochemical staining of 8-oxo-dGuo and β-catenin in *KL* mouse lung tumors at 7 W post Ad-Cre administration. Pearson correlation coefficent (PCC) and *P* value were shown. **h** The *KL* lung cancer cells were treated with indicated ROS inducers (PHEN at 500 μM, PL at 12.5 μM, PEITC at 15 μM) for 6 h and the relative expression of the Wnt pathway-related genes were assessed by real-time PCR. *P < 0.05. **P < 0.01. ***P < 0.001, ****P < 0.0001

According to the bifurcation theory of dynamical systems, the tipping point indicates a state before the imminent critical transition of the system, accompanied by a drastic decline of system resilience, triggering abrupt and irreversible state shift.^31,34,35,42,43^ We then applied this theoretical framework to analyze the AST process (Fig. 2a). The lung tumor system begins with a gradual (reversible) state change in the ADC phase, approaching the tipping point (TP) after which it undergoes a critical (irreversible) state shift, and abruptly switches to the SCC state, which is then further stabilized. This whole process is accompanied by an earlier loss and a later re-gain of system resilience. To quantitatively delineate when and how the critical transition of AST occurred, we employed a previously developed method, dynamic network biomarker (DNB),^36,38–40^ to characterize the critical molecular network of the tipping point. The DNB method designs a composite index (CI) to measure system criticality, and the CI would reach its maximum as the system approaches the tipping point. The DNB analyses revealed a sharp peak at 7 W, indicating a candidate tipping point (Fig. 2b). From the dynamical perspective, the DNB member genes were composed of a strongly fluctuated and highly correlated sub-network specifically at 7 W (Fig. 2c), a time point consistent with our previous analyses (Fig. 1b–d). This was further confirmed by another method with the application of sliding window in silico to artificially increase the number of samples at each time point (Supplementary Fig. 1a). Our data collectively identified the 7 W was the TP in the AST process.

### Wnt signaling is inactivated by ROS at the AST tipping point

We next sought to identify those specifically dysregulated genes across the tipping point (7 W). We found that the DNB molecules were significantly enriched for the Wnt signaling pathway (Fig. 2d). Moreover, this pathway was also enriched in the differential genes before and after 7 W (Supplementary Fig. 1b). Interestingly, we observed a clear fluctuation of the Wnt signaling activity across the tipping point, after which its activity endured a sudden decline (Supplementary Fig. 1c, d). From these TP-induced genes, we found a significant enrichment of known squamous lineage TFs such as *TP63* and *SOX2* (Supplementary Fig. 1e). From the TP-repressed genes, known adenomatous lineage TFs such as *NKX2-1* and *FOXA2* were significantly enriched (Fig. 2e). Such attenuation of adenomatous lineage TFs and simultaneous activation of squamous lineage TFs well corresponded to the critical transition of cellular fates across the tipping point. Moreover, we noticed that three TFs of the Wnt signaling pathway were also among the top dysregulated TFs (Fig. 2e). These data together indicate that the Wnt signaling might be involved in the TP regulation and lineage transition during AST.

We then investigated the potential mechanisms of the Wnt pathway dysregulation. Given previous reports that excessive accumulation of ROS is considered as an important factor to promote AST^29^ and oxidative stress is also known to inhibit the Wnt pathway,^44–46^ we tested the hypothesis that the abrupt inactivation of the Wnt signaling at 7 W might be due to high ROS level. As expected, ROS accumulated and peaked at 7 W after Ad-cre administration in *KL* model (Supplementary Fig. 1f). Through immunostaining of 7 W ADC from *KL* mouse model, we found that β-catenin positivity was negatively correlated with the level of 8-oxo-2’-deoxyguanosine (8-oxo-dGuo), the most common oxidative lesion observed in duplex DNA (Fig. 2f, g). Moreover, treatments with phenformin (PHEN), piperlongumine (PL), and phenethyl isothiocyanate (PEITC) in *Kras*^*G12D*^*; Lkb1*^*-/-*^ (KL) mouse lung ADC cells consistently increased the ROS level (Supplementary Fig. 1g) and subsequently inhibited the Wnt signaling (Fig. 2h). Consistent with previous studies,^44,47^ these ROS inducer treatments significantly increased the *FoxO3a* gene expression (Supplementary Fig. 2a). We found that FOXO3A expression was positively correlated with 8-oxo-dGuo level at 7 W, indicating the upregulation of FOXO3A in response to oxidative stress (Supplementary Fig. 2b). Moreover, β-catenin level was negatively correlated with FOXO3A expression (Supplementary Fig. 2c). Consistently, we found that *FoxO3a* knockout activated the Wnt pathway as indicated by the upregulation of *Ctnnb1* and *Axin2* (Supplementary Fig. 2d–f). Moreover, *FoxO3a* knockout also significantly relieved the inhibition of the Wnt pathway triggered by multiple ROS inducers (Supplementary Fig. 2g). Consistent with a previous study about the de-regulation of fatty acid oxidation in KL SCC,^29^ we found that the ROS inducers promoted the accumulation of lipid-related ROS (Supplementary Fig. 2h).^48^ These data together demonstrate that the Wnt pathway is inactivated by upregulated FOXO3A under the condition of excessive accumulation of ROS.

### Genetic perturbation validates an important role of the Wnt signaling in AST

**Fig. 3:**
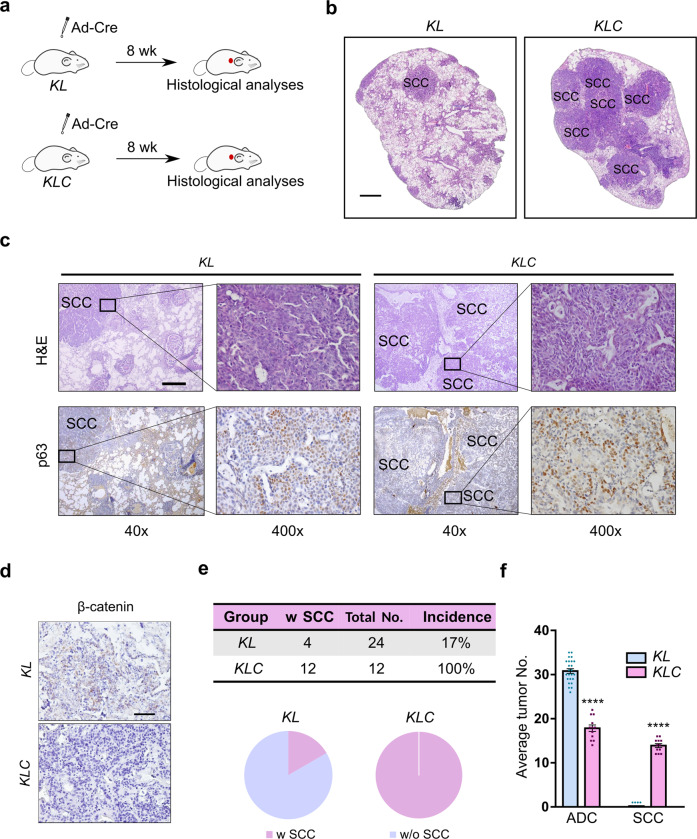
Inactivation of the Wnt pathway promotes the AST process in *KL* mouse model. **a** The *Kras* ^*LSL-G12D/+*^; *Lkb1*^*flox/flox*^ (*KL*, *n* = 24) and *Kras* ^*LSL-G12D/+*^; *Lkb1*^*flox/flox*^*; Ctnnb1*^*flox/flox*^ (*KLC*, *n* = 12) mice were given Ad-Cre via nasal inhalation and histologically analyzed at 8 weeks afterwards. **b** Representative H&E staining on *KL* and *KLC* mouse lung tumors. SCC lesions were indicated. Scale bar: 1 mm. **c** Representative H&E staining and p63 immunohistochemical staining on *KL* and *KLC* mouse lung tumors. SCC lesions were indicated. Scale bar: 500 µm. **d** Representative β-catenin immunohistochemical staining on *KL* and *KLC* mouse lung tumors. Scale bar: 50 µm. **e** SCC incidence of *KL* and *KLC* mice at 8 weeks post Ad-Cre treatment. Tumor No. = Tumor number, w SCC = with SCC, w/o SCC = without SCC. **f**. Average ADC and SCC numbers of *KL* and *KLC* mice at 8 weeks post Ad-Cre treatment. ****P < 0.0001

To test the potential contribution of the Wnt pathway in AST, we performed bidirectional genetic perturbation in mouse models and analyzed their impacts upon the AST process. We first crossed the *Ctnnb1*^*flox/flox*^ mice, in which the conditional deletion of the exons 2–6 inactivates *Ctnnb1* and thus the Wnt pathway,^49,50^ with the *KL* mice to generate the *Kras*^*LSL-G12D/+*^*; Lkb1*^*flox/flox*^*; Ctnnb1*^*flox/flox*^ (*KLC*) cohort. At 8 weeks post Ad-Cre administration, we found a striking increase of SCC development in the *KLC* mice (Fig. 3a–c). As expected, a negative staining of β-catenin was observed in *KLC* mouse tumors (Fig. 3d). All *KLC* mice (100%, 12/12) developed SCC, in contrast to only 17% (4 of 24) incidence of squamous transdifferentiation in the *KL* mice (Fig. 3e). The *Ctnnb1* deletion clearly shifted the tumor transdifferentiation into SCC, with a notable decrease of ADC number (Fig. 3f). These data support an important role of the Wnt inactivation in promoting squamous transdifferentiation.

**Fig. 4:**
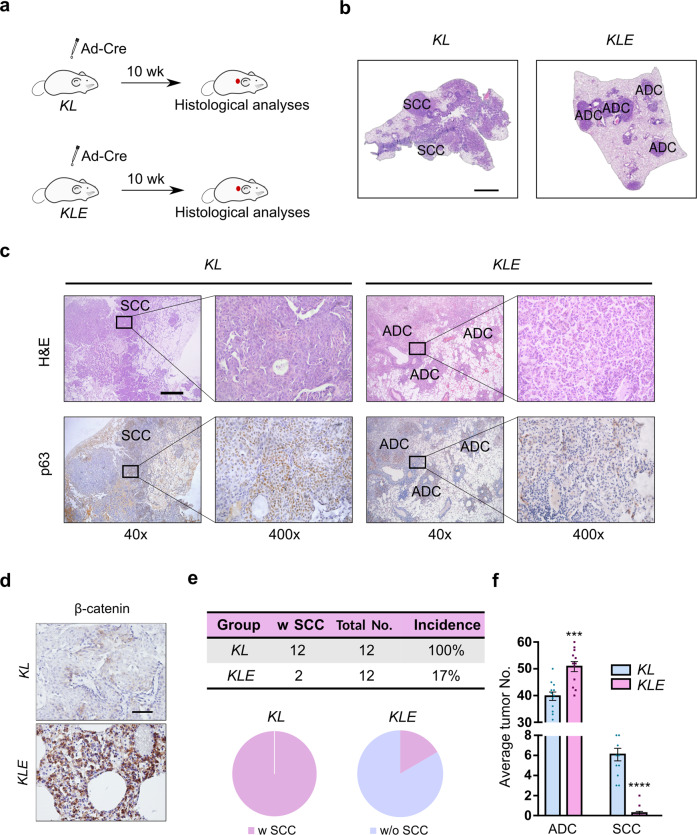
Constitutive activation of the Wnt pathway inhibits the AST process in *KL* mouse model. **a** The *Kras* ^*LSL-G12D/+*^; *Lkb1*^*flox/flox*^ (*KL*) and *Kras* ^*LSL-G12D/+*^; *Lkb1*^*flox/flox*^; *Ctnnb1*^*(E3)flox/flox*^ (*KLE*) mice (*n* = 12 for each group) were given Ad-Cre via nasal inhalation and histologically analyzed at 10 weeks afterwards. **b** Representative H&E staining on *KL* and *KLE* mouse lung tumors. ADC and SCC lesions were indicated. Scale bar: 1 mm. **c** Representative H&E and p63 immunohistochemical staining on *KL* and *KLE* mouse lung tumors. ADC and SCC lesions were as indicated. Scale bar: 500 µm. **d** Representative β-catenin immunohistochemical staining on *KL* and *KLE* mouse lung tumors. Scale bar: 50 µm. **e** SCC incidence of *KL* and *KLE* mice at 10 weeks post Ad-Cre treatment. Tumor No. = Tumor number, w SCC = with SCC, w/o SCC = without SCC. **f** Average ADC and SCC numbers of *KL* and *KLE* mice at 10 weeks post Ad-Cre treatment. ***P < 0.001, ****P < 0.0001

We further investigated whether constitutive activation of the Wnt pathway would inhibit the AST process. For this, we crossed the *Ctnnb1*^*(E3)flox/flox*^ mice, in which the exon3 of β-catenin can be conditionally deleted through Ad-Cre administration for persistent β-catenin nuclear retention and thus the Wnt signaling activation,^51–54^ with the *KL* mice to generate the *Kras*^*LSL-G12D/+*^; *Lkb1* ^*flox/flox*^; *Ctnnb1*^*(E3)flox/flox*^ (*KLE*) cohort for subsequent analyses (Fig. 4a). Histopathological analyses showed that constitutive Wnt activation resulted in a significant decrease of SCC number and incidence in the *KLE* mice (Fig. 4b–e). With the decrease of SCC number, we observed a slight increase of ADC number (Fig. 4f). Taken together, these data support a critical inhibitory role of the Wnt pathway in orchestrating the AST process.

### Pharmacological activation of Wnt signaling inhibits AST only before the tipping point

**Fig. 5:**
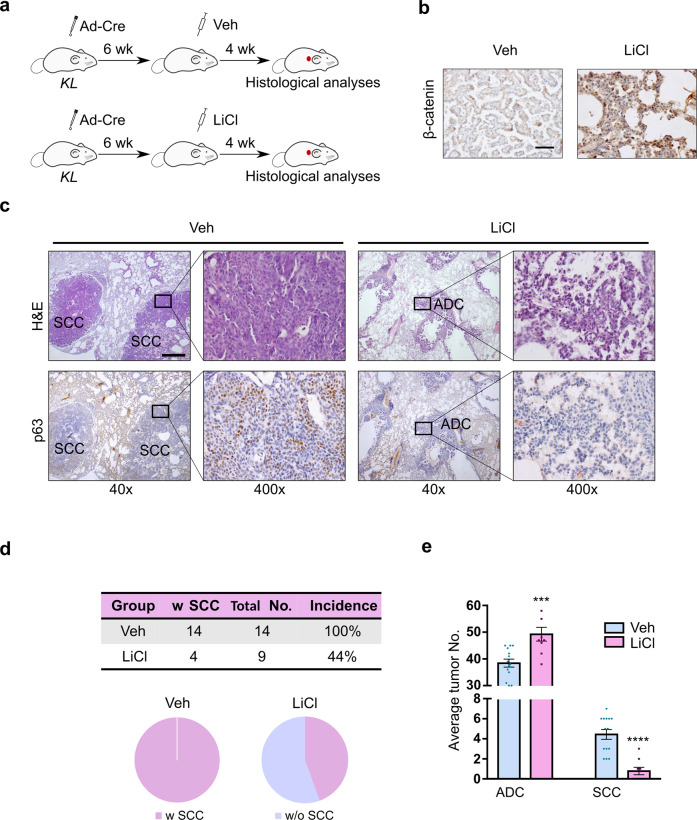
LiCl treatment before the tipping point significantly inhibits the AST process in *KL* mouse model. **a** A scheme of LiCl treatment before the tipping point in *KL* mice (Veh: control group, *n* = 14, LiCl: LiCl treatment group, *n* = 9). **b** Representative β-catenin immunohistochemical staining on *KL* mouse lung tumors with or without LiCl treatment. Scale bar: 50 µm. **c** Representative H&E staining and p63 immunohistochemical staining on *KL* mouse lung tumors with or without LiCl treatment. ADC and SCC lesions were indicated. Scale bar: 500 µm. **d** Quantification of SCC incidence of *KL* mice with or without LiCl treatment at 6 weeks post Ad-Cre treatment. Tumor No. = Tumor number, w SCC = with SCC, w/o SCC = without SCC. **e** Quantification of average tumor numbers in *KL* mice with or without LiCl treatment at 6 weeks post Ad-Cre treatment. ***P < 0.001, ****P < 0.0001

According to the property of the tipping point before a critical transition and the hypothesis justified above, an early blockade of the Wnt signaling attenuation before the tipping point (at a reversible state) would effectively inhibit the AST, whereas a late perturbation after the tipping point (at an irreversible state) might not have such a significant effect. To test this hypothesis, we took advantage of lithium chloride (LiCl), a known β-catenin agonist that activates the Wnt signaling through GSK3 inhibition, to treat *KL* mice at 6 W (before TP) and 10 W (after TP) post Ad-Cre administration (Fig. 5a and Supplementary Fig. 3a). Consistently, we found that LiCl treatment resulted in a significant increase of nuclear β-catenin level (Fig. 5b). Moreover, we found that the early treatment with LiCl (at 6 W) resulted in a notable decrease in both SCC incidence and number (Fig. 5c–e). In stark contrast, the late treatment of LiCl showed almost no impact on SCC incidence and number (Supplementary Fig. 3b, c). We observed a slight increase of average ADC number after late LiCl treatment, presumably due to the promotive function of Wnt signaling upon lung ADC.^55–58^ These data together demonstrate the importance of the Wnt signaling perturbation in regulating AST process and highlight the irreversibility of AST after crossing tipping point.

### Mutual suppressive lineage-specific transcription factor network between ADC and SCC

**Fig. 6:**
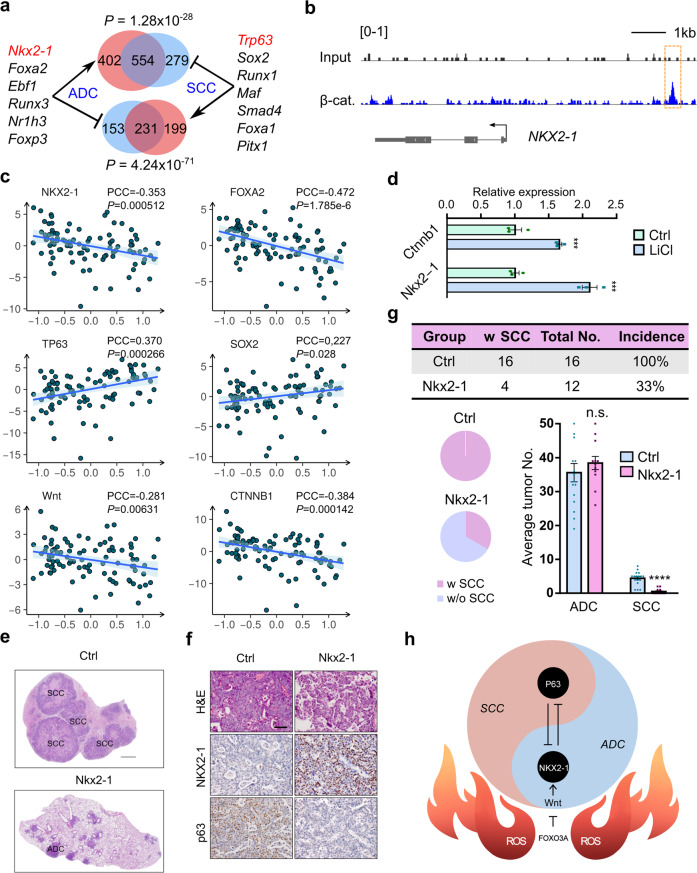
The Wnt-NKX2-1 axis regulates the AST process at the tipping point. **a** Mutually suppressive model of ADC and SCC lineage-specific TF networks based on computation analyses. The significance of overlapped target genes was evaluated with Fisher’s exact test. **b** ChIP-seq data showing the β-catenin binding peak located upstream of human *NKX2-1* allele. β-cat: β-catenin. NCBI SRA accessions: SRX1036445 and SRX833403. **c** A total of 93 human lung adenosquamous carcinoma were used for RNA-seq analyses and aligned individually from the ADC-like state to the SCC-like state based on the AST score. The AST score was defined by the difference between SCC signature and ADC signature based on the TCGA dataset. The correlation between AST score and indicated gene signature was calculated. **d** Mouse *Kras*^*G12D*^*; Lkb1*^*-/-*^ lung cancer cells were treated with LiCl (10 mM) for 24 h and the relative gene expression of *Ctnnb1* and *Nkx2-1* was assessed by real-time PCR. ***P < 0.001. **e** H&E staining of mouse lung tumors from *KLC* mice at 13 weeks post Lenti-Cre-Nkx2-1 nasal inhalation. Scale bar: 1 mm. Ctrl: *KLC* mice (Ctrl: *n* = 16, Nkx2-1: *n* = 12). **f** Representative photos of H&E staining, NKX2-1 and p63 immunohistochemical staining on mouse lung tumors from *KLC* mice post 13 weeks of Lenti-Cre or Lenti-Cre-Nkx2-1 nasal inhalation. Scale bar: 50 µm. Ctrl: *KLC* mice. **g** SCC incidence and average tumor numbers in *KLC* mice post 13 weeks of Lenti-Cre or Lenti-Nkx2-1 nasal inhalation. ****P < 0.0001, n.s. not significant. Ctrl: *KLC* mice. Tumor No. = Tumor number, w SCC = with SCC, w/o SCC = without SCC. **h** Mechanistic model of AST. In this model, the dynamic homeostasis between ADC and SCC lineage-specific transcriptional factors is finely tuned by the Wnt signaling, which maintains the ADC state. At the AST tipping point, excessive ROS accumulation abruptly suppresses the Wnt signaling as mediated by FOXO3A, and leads to the destabilization of adenomatous TF program and eventually push towards the activation of squamous TF program

To decipher the mechanisms involved in TP-related Wnt signaling in the context of AST process, we explored the potential transcriptional modulation involved in this lineage transition. We first computationally analyzed the key transcriptional factor networks in contribution to ADC and SCC development using murine transcriptomic dataset categorized by ADC or SCC phenotype. We found that the ADC transcription factor network was composed of those known ADC lineage factors *Nkx2-1*, *Foxa2*, etc. (Fig. 6a). In contrast, the SCC transcriptional factor network was featured with key TFs such as *p63*, *Sox2*, etc. (Fig. 6a). Notably, two lineage-maintenance regulatory programs existed: the adenomatous program underlying the ADC differentiation, and the squamous program underlying the SCC differentiation. We found these two TF programs were counteracting and exerted their function in a mutual suppressive manner (Fig. 6a). The ADC-lineage TF program specifically drove the induction of genes that were actively tuned down by the SCC-lineage TF program (*P* = 1.28e−28), and vice versa (*P* = 4.24e−71) (Fig. 6a). Using the TCGA datasets, we further found that such counteracting pattern between ADC and SCC-lineage-specific TF programs also existed in human lung cancer (Supplementary Fig. 4a). Moreover, these ADC or SCC-lineage-specific TFs themselves also showed a direct mutual suppressive pattern at transcriptional levels (Supplementary Fig. 4b). These data together uncover a “Yin-Yang” counteracting pattern between mouse and human ADC and SCC TF networks which mutually suppress each other as well as their downstream target genes.

### A mechanistic model for AST orchestrated by the Wnt signaling

We reasoned that the adenomatous program should be switched to the antagonistic squamous program during the AST process and hypothesized that the Wnt signaling might help maintain the adenomatous state. To test this, we first analyzed the ChIP-seq data of β-catenin. Clearly, we can detect the enrichment of the β-catenin binding motif in the gene promoter of the adenomatous lineage factor *NKX2-1* (Fig. 6b). To further solidify this conclusion, we collected a cohort of 93 Chinese lung adenosquamous carcinomas and performed transcriptomic analyses. Although these samples were pathologically confirmed to contain both adenomatous and squamous lesions, some specimens might contain more ADC components whereas others contain more SCC components due to the bias of sample collection. To overcome this issue, we defined an AST score by quantifying the proximity towards the squamous state. Using this score, we could align these samples individually from the ADC-like state to the SCC-like state. Clearly, our analyses showed that the ADC lineage TFs such as *NKX2-1* and *FOXA2* were significantly negatively correlated with the AST score whereas the *TP63* and *SOX2*, two SCC lineage TFs, were opposite (Fig. 6c). Importantly, we found that the signatures for the Wnt signaling were negatively correlated with the AST score (Fig. 6c), similar to the NKX2-1 signature. These data indicate the potential regulation of the Wnt pathway on NKX2-1 gene transcription.

To further solidify this, we treated mouse KL lung ADC cells with LiCl. We found that the activation of the Wnt pathway indeed promoted the *Nkx2-1* gene expression (Fig. 6d and Supplementary Fig. 5a). Treatment with ICG-001, a β-catenin inhibitor,^59–63^ significantly down-regulated *Nkx2-1* expression (Supplementary Fig. 5b, c). Moreover, the inactivation of the Wnt pathway using *Ctnnb1* knockout significantly decreased *Nkx2-1* expression (Supplementary Fig. 5d–i). To investigate whether *Nkx2-1* indeed regulates AST in context with the Wnt signaling, we delivered the lentivirus carrying the expression of Nkx2-1 and Cre (Lenti-*Nkx2-1*-*Cre*) into the *KLC* mice (Supplementary Fig. 4c). We found that, in contrast to control mice, ectopic NKX2-1 expression obviously inhibited the squamous transdifferentiation (Fig. 6e, f). Only about 33% (4/12) mice showed SCC in comparison to 100% SCC incidence in control mice (Fig. 6g). Moreover, the majority of lesions from the Nkx2-1 group were ADC (Fig. 6e–g). Consistently, the SCC number was also reduced in the Nkx2-1 group (Fig. 6g). These data demonstrate that ectopic *Nkx2-1* expression inhibits the AST process accelerated by the Wnt pathway inactivation.

Collectively, we propose a mechanistic model for AST in *KL* mouse model (Fig. 6h). The ADC and SCC differentiation states are respectively controlled by the adenomatous TF program (e.g., *NKX2-1*) and the squamous TF program (e.g., *TP63*). The Wnt signaling functions as a stabilizer of the adenomatous TF program and favors the adenomatous differentiation through the activation of NKX2-1. In the *KL* mice, excessive accumulation of ROS elicits a FOXO3A-mediated switch-off of the Wnt/β-catenin signaling at the tipping point (7 W), leading to the destabilization of the adenomatous TF program which in turn activates the squamous TF program. After a short fluctuation period near the tipping point, the squamous TF program wins out and the system shifts to the squamous differentiation state.

## Discussion

Lung cancer is a highly malignant cancer with strong plasticity. Several clinically important phenomena, such as the occurrence of lung AdSCC and relapse-associated AST, have been consistently detected but remain poorly understood. Using the well-established AST mouse model, we here extensively characterize the temporal transcriptomic dynamics of lung tumors during the AST process from a network perspective and uncover the tipping point. We further identify the Wnt signaling as the critical regulator at the tipping point and propose an ADC and SCC-lineage specific TFs mutual suppressive model. Our previous study has shown that excessive ROS accumulation serves as an important upstream trigger for AST.^29^ However, it remains unknown how this strong ROS level drives AST. Our data demonstrate that excessive ROS indeed results in an abrupt inactivation of the Wnt signaling, which subsequently disrupts the mutually suppressive homeostasis between ADC and SCC-lineage specific TF network via tuning down NKX2-1 level and eventually promotes the squamous transdifferentiation.

Critical transitions and identification of the tipping point, as exemplified in physiological systems, ecological systems, climate systems, and social systems, have become a useful framework in system state monitoring and perturbation.^31,33–35,37,64^ According to the theory of dynamical systems, the system is very difficult to shift back to its original state once it goes across the tipping point. However, when approaching the tipping point, a very small force might be sufficient to push the system forward. Theoretically, early perturbations before the TP are considered to be important. Of course, a better mechanistic understanding of the tipping point would be largely desired. In biological systems, a network-based DNB has been shown to be effective in the identification of tipping points based on high-dimensional data.^36,38–41^ In this study, we have applied the DNB method in a series of temporal transcriptomic data and uncovered the critical tipping point as the 7th week in *KL* AST mouse model. Our LiCl treatment experiments also prove the irreversibility of the tipping point during the AST process. Interestingly, the critical behavior at the tipping point between the adenomatous and the squamous states is like “Yin” and “Yang”, a pair of key categories borrowed from the classical Chinese literature that vividly signifies counteractions and inter-transitions. Indeed, we find that ADC and SCC are characterized by two mutually suppressive TF network and this homeostasis state is disrupted towards the tipping point of AST. Restoring this subtle perturbation before the tipping point could largely prevent the AST process. Besides the *KL* mouse model, other models have also been established for investigating the AST event. For example, the proportion of mice with DNp63^+^ tumor cells increase over time, with 33% at 4 weeks post induction and 100% at 12 weeks in the *Rosa26*^*LSL-Sox2-IRES-GFP*^*;Nkx2-1*^*flox/flox*^*; Lkb1*^*flox/flox*^ (*SNL*) mouse model.^65^ They point out that NKX2-1 loss accelerates adeno-to-squamous transdifferentiation. Similarly, sequential *Kras* activation and *Nkx2-1/Foxa1/2* deletion resulted in squamocolumnar-junction-like tumors as well as discrete SCC lesions in mice.^66^ Their data show that KRAS^G12D^ activated SPC-positive cells have the potential to undergo full squamous transdifferentiation (CK7-negative/CK5-positive) and become well-differentiated keratinizing SCCs. In our study, the positive regulation of Wnt on *Nkx2-1* is essential to stabilize the ADC state. It would be interesting to investigate whether Wnt is the regulation of *Nkx2-1*in above models. Our analyses of 93 human lung adenosquamous carcinomas showed that the signatures for the Wnt signaling were negatively correlated with the AST score, similar to the *NKX2-1* signature. Based on this, we believe that the consistent patterns of the positive regulation of Wnt on *NKX2-1* would be applied to other mouse models. Future studies will be interesting to check this link between Wnt and AST in other emerging models. Another important extension is in the clinical setting of drug-resistance-related AST,^15,16,18^ which is theoretically and practically important for better understanding the AST process and provides ways for early intervention. Nonetheless, we believe this work would be an important early step in elucidating the tipping point and related mechanism during the AST process, and might provide useful clues for future pharmacological intervention.

Our study has also revealed an unprecedented role for the Wnt signaling in regulating cancer plasticity at the tipping point of AST. The Wnt signaling is well known as a developmental regulatory pathway, where it modulates cellular fates in many organs including the lung.^67–69^ The Wnt signaling has also been linked to diseases including multiple types of cancers, such as colon cancer,^70^ liver cancer,^71^ prostate cancer^72^ and lung cancer.^73^ It has been long proposed that the Wnt signaling maintains cancer stemness.^74–76^ In lung ADC mouse models, the Wnt signaling is often beneficial to tumor growth. Intriguingly, we find that the Wnt signaling can actually specify various cellular fates in the context of lung ADC through stabilizing the adenomatous differentiation state. This provides a very primitive clue that embryonic cellular fate decisions and cancer cell identity might actually be controlled by shared molecular mechanisms. Moreover, the mutually repressive model orchestrated by the Wnt signaling constitutes a transcriptional circuit, which is also highly comparable to classical developmental circuits such as the pluripotency network in stem cells.^77,78^ Wnt signaling is the most significantly enriched pathway in the DNB module whereas other enriched pathways such as Hedgehog pathway^79^ and amino acid metabolism pathways^80^ might also regulate the tipping point. Consistent with our previous studies,^28,29,44,47^ excessive ROS accumulation, e.g., lipid-related ROS, remains as an upstream trigger for such phenotypic transition mediated by the abrupt inactivation of the Wnt signaling and ADC lineage TF dysfunction. Consistent with previous studies,^44,47^ FOXO3A appears to be a major factor involved in the ROS-regulated Wnt pathway dynamics.

It’s worth noting that a recent study on squamous transitioned lung cancer indicates a potential role of Wnt signaling in promoting AST,^20^ e.g., Wnt pathway is upregulated in transitioned lung cancer after Osimertinib resistance. Since this study mainly compares the Wnt pathway between two time points, before and after TKI resistance, it might not be able to uncover the dynamic changes of Wnt signaling. In contrast, we take advantage of the RNA-Seq data from mouse lung tumors at a series of time points during AST process for the pathway analyses. This allows us to detect the dynamic and delicate fluctuations of defined pathway. Our study reaches the consensus that the Wnt signaling is transiently disrupted at the tipping point of AST and such delicate fluctuation is further proven to be important for the AST accomplishment in GEMM. Taken together, our current study has uncovered the signaling importantly for orchestrating the tipping point before AST. Future efforts are necessary to elucidate the whole picture of transcriptional and epigenetic pathways dynamically involved in the AST process.

The epigenetic regulatory mechanism underlying the AST process is another important yet less explored topic. We and collaborators have previously revealed the contribution of Polycomb Repressive Complex 2 (PRC2) in regulating the AST process in *KL* mouse model.^23^ The chromatin analysis reveals the loss of H3K27me3 and gain of H3K27ac and H3K4me3 at squamous lineage genes, including *Sox2*, *ΔNp63* and *Ngfr*. Our current work shows an important role of the Wnt signaling in regulating the expression of the adenomatous lineage gene *Nkx2-1*. Moreover, LKB1 inactivation not only promotes AST but also contributes to lung cancer metastasis, which is like two faces of LKB1. LKB1-deficient lung tumors have a SOX17-mediated epigenetic reprogramming to substantiate metastasis.^81^ Such metastatic regulation involves epigenetic mechanisms through modulating chromatin accessibility and enhancing SOX17-targeted transcription. This study has implications for the epigenetic modulation of metastasis in LKB1-deficient lung tumors. Whether such epigenetic modulation contributes to AST would require future studies.

## Materials and methods

### Ethical approval

The research performed in the present study complies with all ethical regulations. Clinical studies were approved by the institutional review board of Shanghai Cancer Hospital, Fudan University. Written informed consent was obtained from all patients (ethical approval no. IRB 2008223-9). Mice were housed in a specific-pathogen-free environment at the Shanghai Institute of Biochemistry and Cell Biology, and treated in accordance with protocols conforming to the guidelines and approved by the Institutional Animal Care and Use Committee of the Shanghai Institutes for Biological Sciences, Chinese Academy of Sciences (ethical approval no. IBCB0011).

### Mouse cohorts

*Kras* ^*LSL-*G12D*/+*^, *Lkb1*^*flox/flox*^, *Ctnnb1*^*flox/flox*^ and *Ctnnb1*^*(E3)flox/flox*^ mice were originally generously provided by Drs. Tyler Jacks, Ronald A. DePinho, and Lijian Hui, respectively. After Ad-Cre administration, the *Ctnnb1*^*flox/flox*^ mice had the deletion of exon 2 to 6 and thus the inactivation of the Wnt signaling.^49,50^ Similarly, the *Ctnnb1* ^*(E3)flox/flox*^ mice had exon 3 deletion after Ad-Cre administration, which resulted in persistent nuclear retention of β-catenin and thus the constitutive activation of the Wnt signaling.^51–54^ All mice were housed in a pathogen-free environment at Shanghai Institute of Biochemistry and Cell Biology and treated in strict accordance with protocols approved by the Institutional Animal Care and Use Committee of the Shanghai Institute of Biochemistry and Cell Biology, Chinese Academy of Sciences. Mice were treated with adenovirus or lentivirus carrying Cre recombinase (2 × 10^6^ pfu) via nasal inhalation as previously described.^27^ For temporal transcriptomic analyses, the *Kras* ^*LSL-*G12D*/+*^*; Lkb1*^*flox/flox*^ (*KL*) mice were given Ad-Cre through nasal inhalation and sacrificed at a series of time points (0 W, 4 W, 6 W, 7 W, 8 W, 9 W, 10 W) for tumor analyses. The *Kras* ^*LSL-*G12D*/+*^; *Lkb1*^*flox/flox*^*; Ctnnb1*^*flox/flox*^ (*KLC*) mice and control mice were analyzed at 8 weeks post Ad-Cre nasal inhalation. The *Kras* ^*LSL-*G12D*/+*^; *Lkb1* ^*flox/flox*^; *Ctnnb1*^*(E3)flox/flox*^ (*KLE*) mice and control mice were analyzed at 10 weeks post Ad-Cre nasal inhalation. The *KLC* mice were also given Lenti-*Nkx2-1*-*Cre* and analyzed at 13 weeks afterwards. For pharmacological treatment, the *KL* mice were treated with LiCl (500 mg/kg) via intraperitoneal injection every two days for 4 weeks at 6 weeks or 10 weeks post Ad-Cre administration.

### Lentivirus production and infection

The ORFs of *Nkx2-1* and N-terminal truncated β-catenin were amplified from mouse tumor cDNAs, and ligated into expression vectors pCDH-EF1-Puro (Systems Biosciences) or pCDH-EF1-Cre (adapted vector for *Nkx2-1* expression in vivo). The production of lentivirus supernatant and cell infection were described previously.^27^

### Histology and immunohistochemistry

Mouse lungs were inflated with 1 ml 4% paraformaldehyde (PFA), fixed overnight and dehydrated in ethanol, embedded in paraffin, sectioned at 5 μm followed by staining with haematoxylin and eosin (H&E). Photos were taken using Olympus BX53 and Axio Scan.Z1 (Carl Zeiss IMT Co. Ltd.) and tumor numbers were measured using Image J software. The numbers of ADC and.SCC were counted as previously described.^82^ Immunohistochemistry (IHC) was performed as previously described.^27^ The IHC scores of FOXO3A, β-catenin and 8-oxo-dGuo expression were measured as previously described.^83^ Briefly, staining intensities were assigned into 4 grades (intensity score) as follows: 0, negative; 1, low; 2, medium; and 3, high. The IHC score was the sum of intensity score multiplied by percentage. The following antibodies were used: β-catenin (8480, Cell Signaling Technology, 1:500), p63 (ab124762, abcam, 1:1000), SOX2 (ab92494, abcam, 1:500), NKX2-1 (ab133638, abcam, 1:500), SFTPC (AB3786, Millipore, 1:1000), 8-oxo-dGuo (ab48508, abcam, 1:250), FOXO3A (12829, Cell Signaling Technology, 1:250).

### Cell lines and drug treatment

The *Kras*^*G12D*^*;Lkb1*^*-/-*^ (KL) lung cancer cell line was established as previously described^29^ and cultured in DMEM (Hyclone) supplemented with 10% FBS (Biochrom). For LiCl treatment, cells were exposed to LiCl (10 mM) for 24 h and total RNA was extracted for real-time PCR analyses. For ICG-001 treatment, cells were exposed to ICG-001 (1.5 μM) for 48 h and total RNA was extracted for real-time PCR analyses. ICG-001 was purchased from MedChemExpress (HY-14428) and diluted in Dimethyl Sulfoxide (DMSO). For ROS inducer treatment, cells were exposed to PHEN (500 μM) or PL (12.5 μM) or PEITC (15 μM) for 6 h and the total RNA was extracted for real-time PCR analyses. Based on 3-(4, 5-Dimethylthiazol-2-yl)-2, 5-diphenyltetrazolium bromide (MTT) results, we used the indicated concentration.

### RNA extraction and real-time PCR analyses

Mouse lung tumors were freshly dissected at indicated time points post Ad-Cre treatment. Total RNA and genomic DNA from mouse lung tumors and cell lines were prepared as previously described.^27^ Briefly, total RNA was extracted from cells using Trizol reagent (Invitrogen) and reverse transcribed with the PrimeScript RT Reagent Kit (Takara). The cDNAs were then used for real-time PCR on a LightCycler®480/96 Real-Time PCR System (Roche) using SYBR-Green Master PCR mix (Toyobo). β-actin served as internal control. The primers used for real time PCR were:

Mouse *β-Actin* forward: 5′-TGAGCGCAAGTACTCTGTGTGGAT-3′

Mouse *β-Actin* reverse: 5′-ACTCATCGTACTCCTGCTTGCTGA-3′

Mouse *Ctnnb1* forward: 5′-ATGGAGCCGGACAGAAAAGC-3′

Mouse *Ctnnb1* reverse: 5′-TGGGAGGTGTCAACATCTTCTT-3′

Mouse *Nkx2-1* forward: 5′-CAGCGCTTCGGGCCCCGGAT-3′

Mouse *Nkx2-1* reverse: 5′-AGCGAGCCCAGGCCGCCCAT-3′

Mouse *Axin2* forward: 5′-ATGAGTAGCGCCGTGTTAGTG-3′

Mouse *Axin2* reverse: 5′-GGGCATAGGTTTGGTGGACT-3′

Mouse *Ccnd1* forward: 5′-GCGTACCCTGACACCAATCTC-3′

Mouse *Ccnd1* reverse: 5′-ACTTGAAGTAAGATACGGAGGGC-3′

Mouse *c-Myc* forward: 5′-ATGCCCCTCAACGTGAACTTC-3′

Mouse *c-Myc* reverse: 5′-GTCGCAGATGAAATAGGGCTG-3′

Mouse *FoxO3a* forward: 5′-AACGGCTCACTTTGTCCCA-3′

Mouse *FoxO3a* reverse: 5′-TTGATGATCCACCAAGAGC-3′.

### Human lung cancer specimen collection

A total of 93 human lung adenosquamous carcinomas were collected from 2007 to 2016 with the approval of the institutional review board of Shanghai Cancer Hospital, Fudan University. All patients gave written informed consent. Samples were snap-frozen in liquid nitrogen at the time of resection and stored at −80 °C until the mRNA extraction.

### RNA-seq analyses for mouse and human tumor samples

RNA extraction, reverse transcription, library preparation, and quality control were performed following the Illumina standard protocol. Sequencing of the libraries was performed on an Illumina HiSeq sequencing machine, generating paired-end reads (150 bases x 2). Clean reads were obtained after trimming adapters and filtering low-quality reads. The clean reads were mapped to reference genomes (mm10 and hg38) with the STAR aligner and subsequently quantified with the htseq-count tool. A normalization factor was estimated using the RLE method as implemented in edgeR. After normalizing the effective library size, the expression data in reads per million (RPM) was further log2 transformed for downstream analyses.^84^

### Temporal transcriptomic analyses

The temporal transcriptomic data were dimensionally reduced with the principal component analyses (PCA) method. The components were ranked with proportions of variances explained and the first two principal components were used for visualization of the temporal trajectory. Relative expression of selected marker genes was mapped onto the two-dimensional PCA map to examine their respective fluctuations along the trajectory.

### Dynamic network biomarker and tipping point analyses

The dynamic network biomarker (DNB) framework was used to delineate the network dynamics for the tipping point analyses. Briefly, we unraveled the network dynamics within a subspace constrained by the differential genes (*n* = 3092) between the adenomatous and squamous lineages, which are computed from the TCGA cohorts by fitting genewise negative binomial models and the quasi-likelihood F-tests, as implemented in the edgeR package. For each time point with replicates, an estimate of Pearson’s correlation coefficient (PCC) was performed for every gene pair. Absolute values of PCCs were taken as edge strength in these temporal networks. Network communities were detected with the deterministic hierarchical clustering algorithm. As shown by the DNB method, when the system state approaches a tipping point, a group of genes or DNB (module) would emerge, which satisfies the following three conditions simultaneously: (1) the average absolute correlation of any two genes in the group (PCC_i_) increases; (2) the average absolute correlation between genes in and outside of this group (PCCo) decreases; (3) the average standard deviation (SD_i_) highly increases in the group. The composite index (CI) of criticality was thus a combination of these three criteria:^36,40,85^

$${\rm{CI}} = \frac{{\rm{PCC}_i}}{{\rm{PCC}_o}} \ast \rm{SD}_i$$

The module with maximum CI score was chosen as the dominant group of genes, with its CI score depicting the dynamic changes of system criticality, and the time point of maximum CI considered as a reasonable approximation of the tipping point. This theoretical result means that the appearance of a group of collectively fluctuating genes among the observed high-dimensional data signals an imminent critical transition, or briefly, strongly collective fluctuation implies an imminent state transition from one state to another. For a full derivation, simulation and subsequent validations of the DNB method for critical analyses, please refer to the previous publications.^36,38–41,86^

### Analyses of the Wnt signaling and transcriptional signatures

The Wnt signaling pathway and β-catenin signatures were obtained from MSigDB (v6.0), which are the upregulated signatures trained from two experimental datasets (“WNT UP.V1”, “FEVR CTNNB1 TARGETS”). The signature scores are computed using the z-score method implemented in GSVA (v1.26.0).

Transcriptional targets for *TP63*, *SOX2*, *NKX2-1* and *FOXA2* in three mammalian species (human, mouse and rat) were compiled from IPA (Ingenuity Pathway Analysis, Qiagen). For each TF, the expression of the target genes was used to compute the signature score using the z-score method from GSVA (v1.26.0).

The AST signature was based on top differential genes for ADC and SCC computed using the quasi-likelihood method from edgeR v3.20.9 (fold change > 2 and adjusted *P* value < 0.001). An ADC score was calculated as the average of standardized expression for genes upregulated in ADC. An SCC score is similarly calculated using the genes upregulated in SCC. The final AST score is defined as the difference between the two scores in each sample (AST score = SCC score−ADC score).

### Statistical analyses

Differences were compared using the two-tailed Student’s *t* test, with *P* value < 0.05 considered statistically significant. All analyses were performed with Graphpad Prism 7.

## Supplementary information

Supplementary data

Additional raw data: unprocessed immuneblot data

## Data Availability

The mouse KL lung cancer and human AdSCC datasets have been deposited in the NODE database (project accessions OEP002019 and OEP001032). The materials used in this study are available from the corresponding authors upon reasonable request.
