# Supplementary material for: Oxidative stress-triggered Wnt signaling perturbation characterizes the tipping point of lung adeno-to-squamous transdifferentiation
Source: Signal Transduct Target Ther. 2023 Jan 11;8:16. doi: 10.1038/s41392-022-01227-0 (PMC9832009; doi:10.1038/s41392-022-01227-0)
Supplement: Supplementary file 1 — Supplementary data [file 41392_2022_1227_MOESM1_ESM.docx]

Supplementary Materials for

Oxidative stress-triggered Wnt signaling perturbation characterizes the tipping point of lung adeno-to-squamous transdifferentiation

Zhaoyuan Fang^1,2,3,^*, Xiangkun Han^1,2,^*, Yueqing Chen^1,2,^*, Xinyuan Tong^1,2^, Yun Xue^1,2^, Shun Yao^1,2^, Shijie Tang^1,2^, Yunjian Pan^4,5^, Yihua Sun^4,5^, Xue Wang^1,2^, Yujuan Jin^1,2^, Haiquan Chen^4,5^, Liang Hu^1,2^, Lijian Hui^1,2^, Lin Li^6,7,8^, Luonan Chen^1,2,6,8,#^, Hongbin Ji^1,2,6,8,#^

Correspondence to: Hongbin Ji, [hbji@sibcb.ac.cn](mailto:hbji@sibcb.ac.cn); Luonan Chen, lnchen@sibs.ac.cn

**This PDF file includes:**

Materials and Methods

Supplementary Fig 1 to 5

Materials and Methods

**MTT assay**

For 3-(4, 5-Dimethylthiazol-2-yl)-2, 5-diphenyltetrazolium bromide (MTT) assays, KL cells were seeded in 96-well plate (1,500 cells/well) with 5 repeats and treated with indicated concentrations of drugs after 24 hours. The relative cell growth was determined after 72 hours by multiskan spectrum microplate spectrophotometer. Briefly, 20 μL of MTT working solution (5 mg/mL) was added into each well and incubated at 37°C for 4 hours. The supernatants were then removed, and the resultant MTT formazan was dissolved in 100 μL of DMSO. The absorbance was measured at the wavelengths of 570 and 630 nm.

**Measurement of ROS**

Intracellular oxidative stress was assessed by the measurement of intracellular oxidation of 2,7 -dichlorofluorescin (DCFH) (S0033, Beyotime) according to the manufacturer’s instructions. KL cells were seeded into 12-well plate with 3 repeats and treated with PHEN (500 μM), PL (12.5 μM), PEITC (15 μM) the next day for 6 hours before subjected to DCFH staining. Fluorescence of the oxidized form of DCFH was measured using a flow cytometer CytoFlex3 (Beckman Coulter).

Lipid peroxidation levels were measured by BODIPY 581/591 C11 dye (Invitrogen, D3861) according to the manufacturer’s instructions. KL cells were seeded into 12-well plate with 3 repeats and treated with PHEN (500 μM), PL (12.5 μM), PEITC (15 μM) the next day for 6 hours. Then cells were incubated in FBS-free medium containing 5 μM BODIPY 581/591 C11 dye. After incubation at 37℃ for 30 mins, cells were washed and trypsinized and then subjected to flow cytometry analyses.

**Western blot and immunofluorescence staining**

For western blot, whole-cell lysates were prepared in loading buffer (10% SDS, 1 mM DTT and glycerin) and incubated at 100°C for 10 min. Equal volumes of proteins were resolved by SDS–PAGE and transferred onto the PVDF membranes. After incubation in blocking buffer (50 mM Tris-buffered saline [pH 7.4] containing 5% non-fat dry milk and 0.1% Tween-20), the membranes were probed with the primary antibodies, followed by incubation with HRP-linked goat anti-rabbit IgG (CST, 7074S, 1:5000 dilution). Bands were revealed with an ECL kit (Thermo Fisher Scientific) prior to detection on SAGECREATION (Sage Creation Science Co, Beijing).

For immunofluorescence staining, KL mouse lung cancer cell lines with or without sgFoxO3a were seeded into 24-well plate with steriled roud slides. After 24 hours, cells were treated with or without PHEN (500 μM), PL (12.5 μM), PEITC (15 μM) in FBS-free medium for 48 hours. Cells were washed with PBS and fixed with 4% PFA solution. After incubating with primary antibodies overnight in 4℃, cells were washed with PBS for 3 times and incubated with secondary antibodies (Alexa Fluor® 594 Conjugated Anti-rabbit IgG for FOXO3A and Alexa Fluor^®^ 488 Conjugated Anti-rabbit IgG for β-catenin) for 1 hour at room temperature. Then cells were wased with PBS for 3 times and incubated with DAPI for 15 minutes at room temperature. After that cells were washed and fixed for pictures.

Following primary antibodies were used: FOXO3A (2071, EPITOMICS, 1:2,000 dilution for Western blot, 1:100 dilution for immunofluorescence staining), β-catenin (8480, Cell Signaling Technology, 1:5,000 dilution for Western blot, 1:100 dilution for immunofluorescence staining), and TUBULIN (66240-1, proteintech, 1:5,000 dilution)

**Plasmid constructs and virus production**

All sgRNAs were cloned into the LentiCRISPR v2 vector with a puromycin-selection marker. Puromycin was purchased from Sigma. Plasmids were packaged into lentiviral particles by co-transfection with packaging plasmids into HEK293T cells and the filtered cell culture supernatant was then used to infect cells.

sg*Ctnnb1-1* forward: 5’-CACCG CGGGCAGTATGCAATGACTA-3’

sg*Ctnnb1-1* reverse: 5’-AAAC TAGTCATTGCATACTGCCCG C-3’

sg*Ctnnb1-2* forward: 5’-CACCG GATTAACTATCAGGATGACG-3’

sg*Ctnnb1-2* reverse: 5’-AAAC CGTCATCCTGATAGTTAATC C-3’

sg*Ctnnb1-3* forward: 5’-CACCG ATGAGCAGCGTCAAACTGCG-3’

sg*Ctnnb1-3* reverse: 5’- AAAC CGCAGTTTGACGCTGCTCAT C-3’

sg*FoxO3a* forward: 5’-CACCG CGTTGGAATTGGTGCGCGAG-3’

sg*FoxO3a* reverse: 5’- AAAC CTCGCGCACCAATTCCAACG C-3’

sg*Control* forward: 5’- CACCG GGCCACGAGTTCGAGATCGA-3’

sg*Control* reverse: 5’- AAAC TCGATCTCGAACTCGTGGCC C-3’

**Identification of lineage-specific transcription factor networks**

A compendium of human transcription factors (TFs) was compiled by taking the union of these two representative databases: AnimalTFDB and TCoF. Candidate target genes for transcription factors were predicted based on TF binding data from the ChIP-Atlas datasets, based on the criterion that at least one binding site of a TF within 1kb of the transcription start sites (TSSs) for its candidate target genes. Given gene expression cohorts of lung ADC and SCC, we refined the target genes by enforcing conditional co-expression between each TF and its candidate target genes. Practically, the candidate genes ranking top 1000 by positive (or negative) co-expression with each TF in either cohort were retained for further analyses. Thus we obtained both induced and repressed target genes for each TF, conditionally. ADC- (or SCC-) specific TFs were defined as those most significantly over-expressed in ADC (or SCC) (Pearson’s correlation test, *P* < 1 x 10^-20^). With these lineage-specific TFs and their conditional target genes, we built the key TF networks using these regulatory links for ADC and SCC, respectively.

**
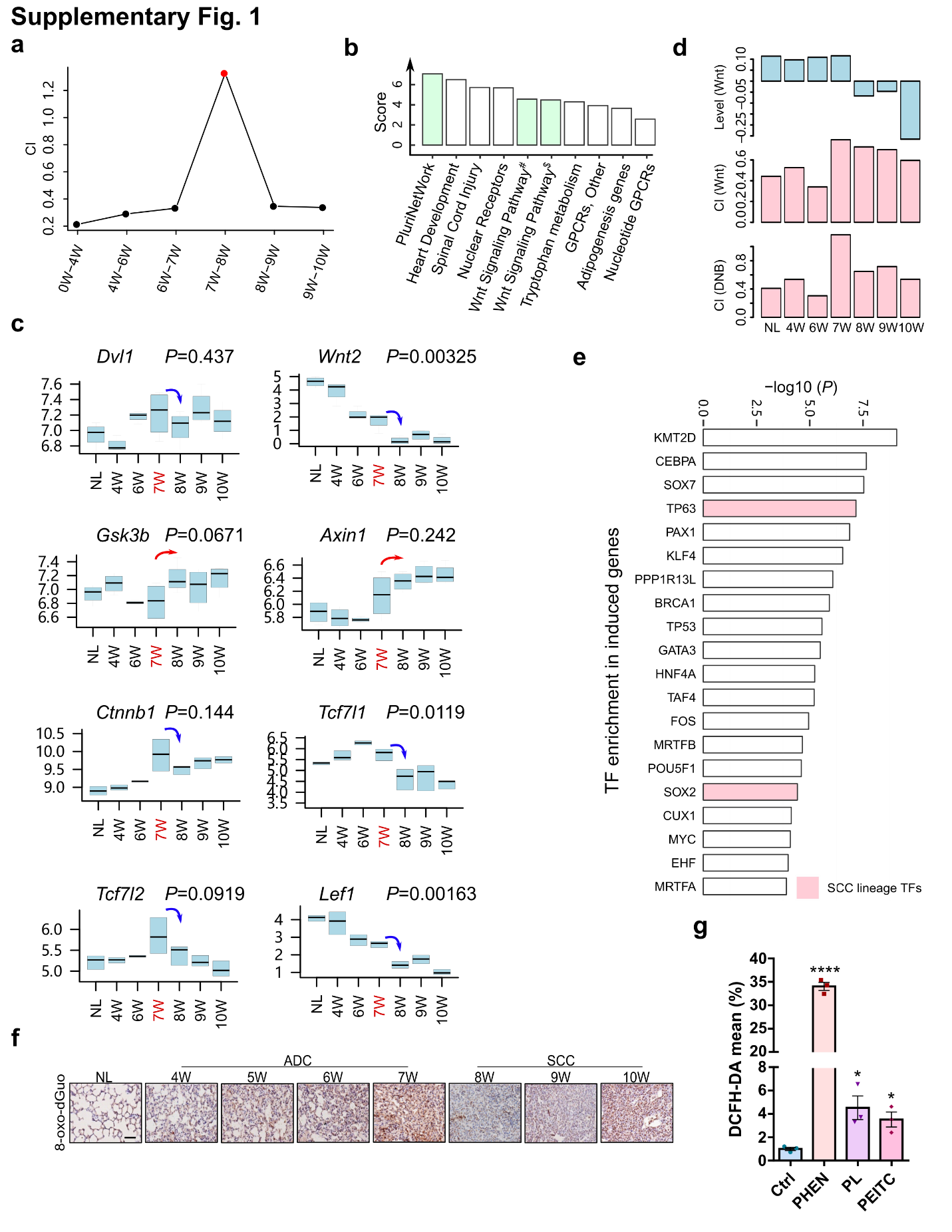
Supplementary Figures**

**Supplementary Fig 1. Wnt/β-catenin pathway dysregulation in the tipping point of AST.**

**a**. Composite index (CI) after sliding window analyses. Each time point is composed of two neighboring weeks. The peak of CI at 7W-8W indicates the tipping point.

**b**. Biological pathways enrichment in deregulated genes across the tipping point. Top significant pathways from WikiPathways database (https://www.**wikipathways**.org) were ranked by the enrichment score. ^#^WP539 Wnt signaling pathway (106 genes); ^$^WP403 Wnt signaling pathway (58 genes).

**c**. Dynamic expression changes of Wnt/β-catenin pathway genes. The edgeR differential expression test *P* values are also labeled (7W versus 8W).

**d.** Wnt/β-catenin signature level, and its corresponding CI score, together with the DNB CI score.

**e**. Transcription Factor (TF) enrichment in the upstream of genes induced across the tipping point. Top 20 TFs are ranked with -log10(*P* value) and the SCC lineage TFs were highlighted.

**f**. Representative immunohistochemical staining of 8-oxo-dGuo of *KL* mouse lungs at a serial of time points after Ad-Cre nasal inhalation. NL: normal lung. Scale bar: 50 µm.

**g.** ROS levels in mouse KL cells under indicated culture conditions (PHEN at 500 μM, PL at 12.5 μM, PEITC at 15 μM) for 6 hours. Results are expressed as the percentage change in the mean DCFH-DA values. Data are shown as mean ± SEM. **P <* 0.05, *****P <* 0.0001.


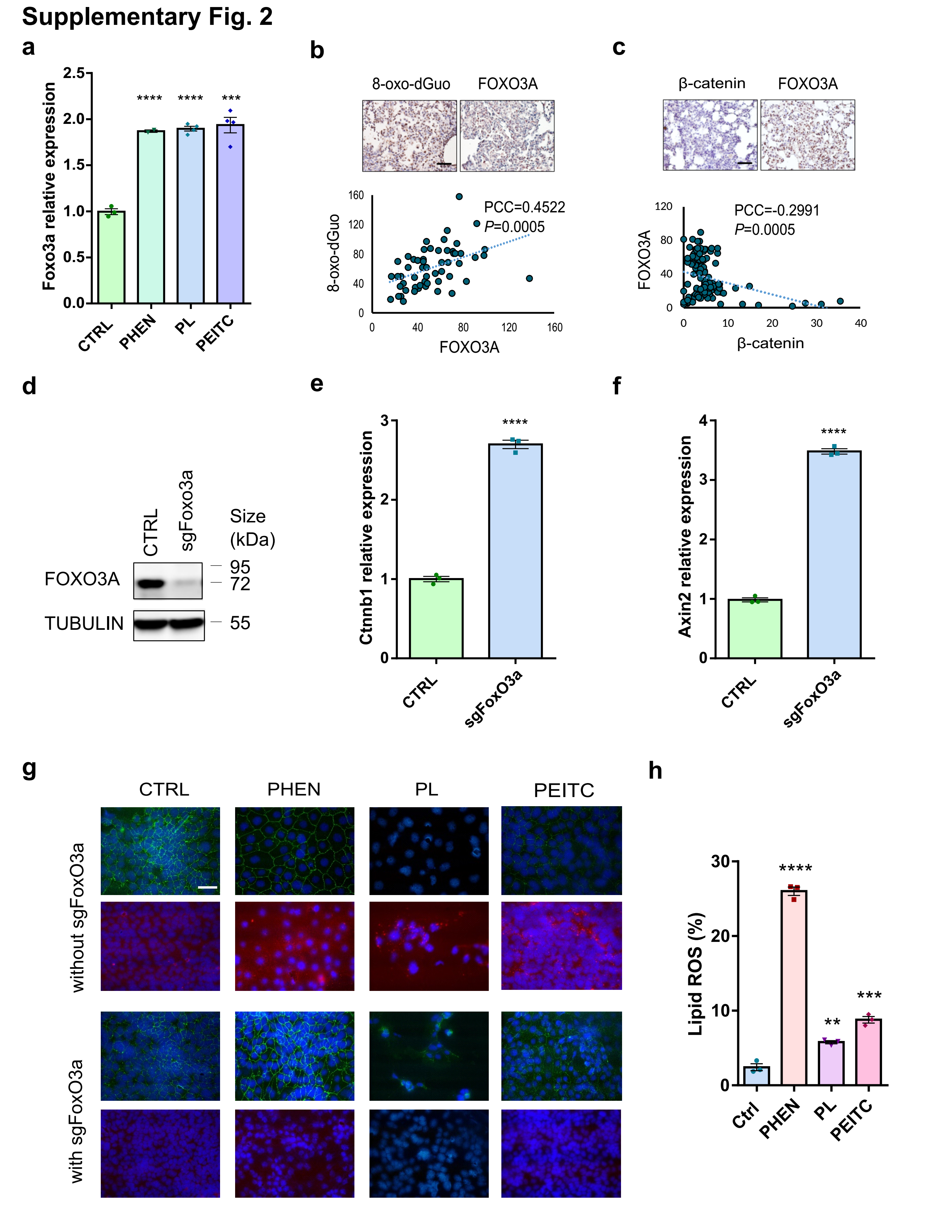
**Supplementary Fig 2. FOXO3A regulates β-catenin under ROS induction.**

**a**. Real-time PCR quantification of *FoxO3a* gene expression in mouse KL cell line with or without ROS inducer. Data are shown as mean ± SEM. ****P <* 0.001, *****P <* 0.0001.

**b**. Representative photos for immunohistochemical staining of FOXO3A and 8-oxo-dGuo in KL mouse lung tumors at 7 weeks post Ad-Cre treatment. Scale bar: 50 µm. Related correlation of immunohistochemical staining of FOXO3A and 8-oxo-dGuo was also shown. PCC: Pearson’s correlation coefficient.

**c**. Representative photos for immunohistochemical staining of FOXO3A and β-catenin in KL mouse lung tumors at 7W post Ad-Cre treatment. Scale bar: 50 µm. Related correlation of immunohistochemical staining of FOXO3A and β-catenin was shown.

**d**. Western blotting analyses of FOXO3A levels in mouse KL cell line with or without sg*FoxO3a*. TUBULIN serves as the control.

**e**. Real-time PCR quantification of *Ctnnb1* gene expression in mouse KL cell line with or without sg*FoxO3a*. Data are shown as mean ± SEM. *****P <* 0.0001.

**f**. Real-time PCR quantification of *Axin2* gene expression in mouse KL cell line with or without sg*FoxO3a*. Data are shown as mean ± SEM. *****P <* 0.0001.

**g**. Immunofluorescence analyses of p-FOXO3A (red) and β-catenin (green) protein levels of mouse KL cell line under or without ROS inducer with or without sg*FoxO3a*. Scale bar: 50 µm.

**h.** Lipid ROS levels in mouse KL cells under indicated culture conditions (PHEN at 500 μM, PL at 12.5 μM, PEITC at 15 μM) for 6 hours. Results are detected by BODIPY 581/591 C11 dye. Data are shown as mean ± SEM. ***P <* 0.01, ****P <* 0.001, *****P <* 0.0001.

**
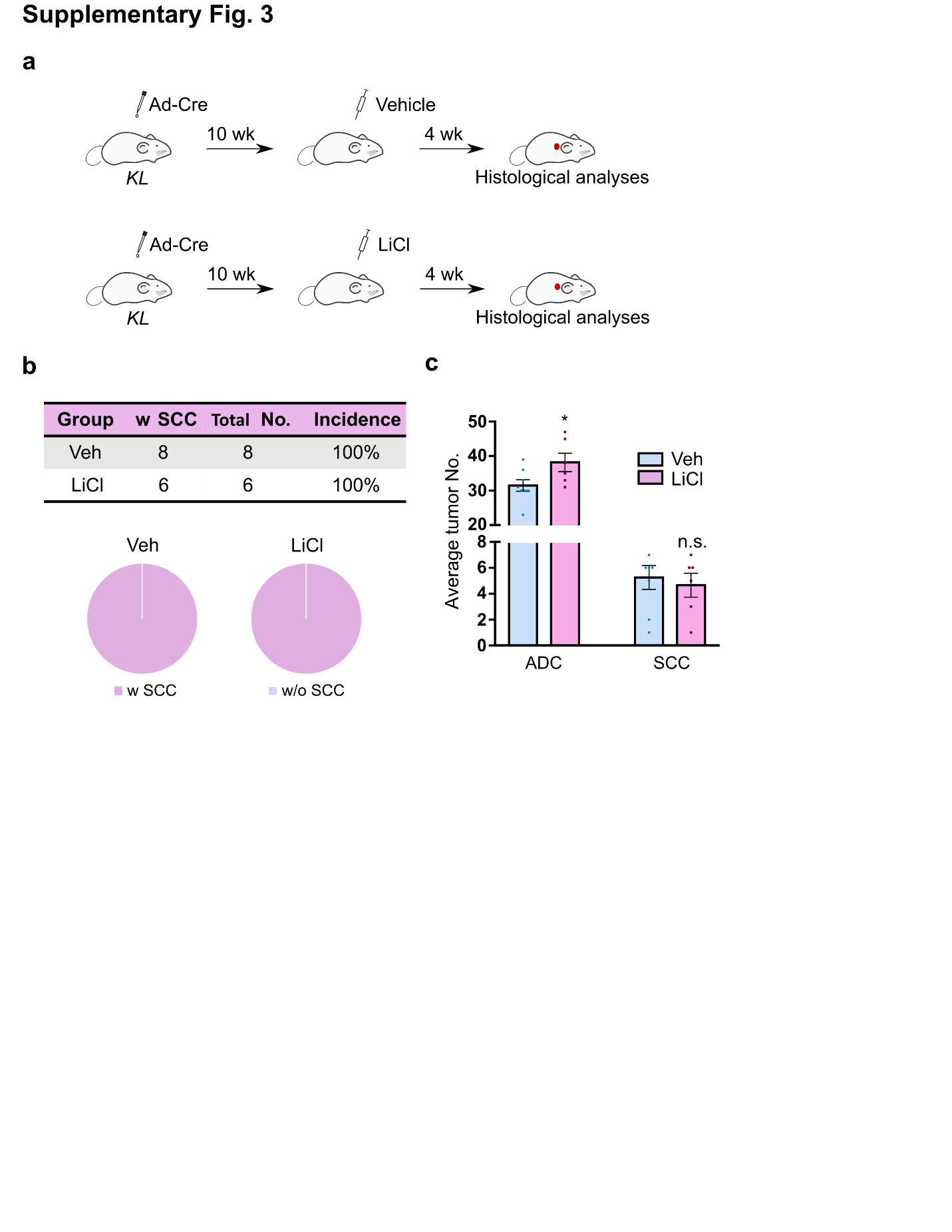
**

**Supplementary Fig 3. LiCl treatment after the tipping point.**

**a**. A scheme of LiCl treatment after the tipping point in *KL* mice (Veh: control group, n=8; LiCl: LiCl treatment group, n=6).

**b**. Quantification of SCC incidence of *KL* mice with or without LiCl treatment at 10 weeks post Ad-Cre treatment. Tumor No. = Tumor number, w SCC = with SCC, w/o SCC = without SCC.

**c*.*** Quantification of average tumor numbers in *KL* mice with or without LiCl treatment at 10 weeks post Ad-Cre treatment. **P <*0.05, n.s. not significant (Student’s t test).


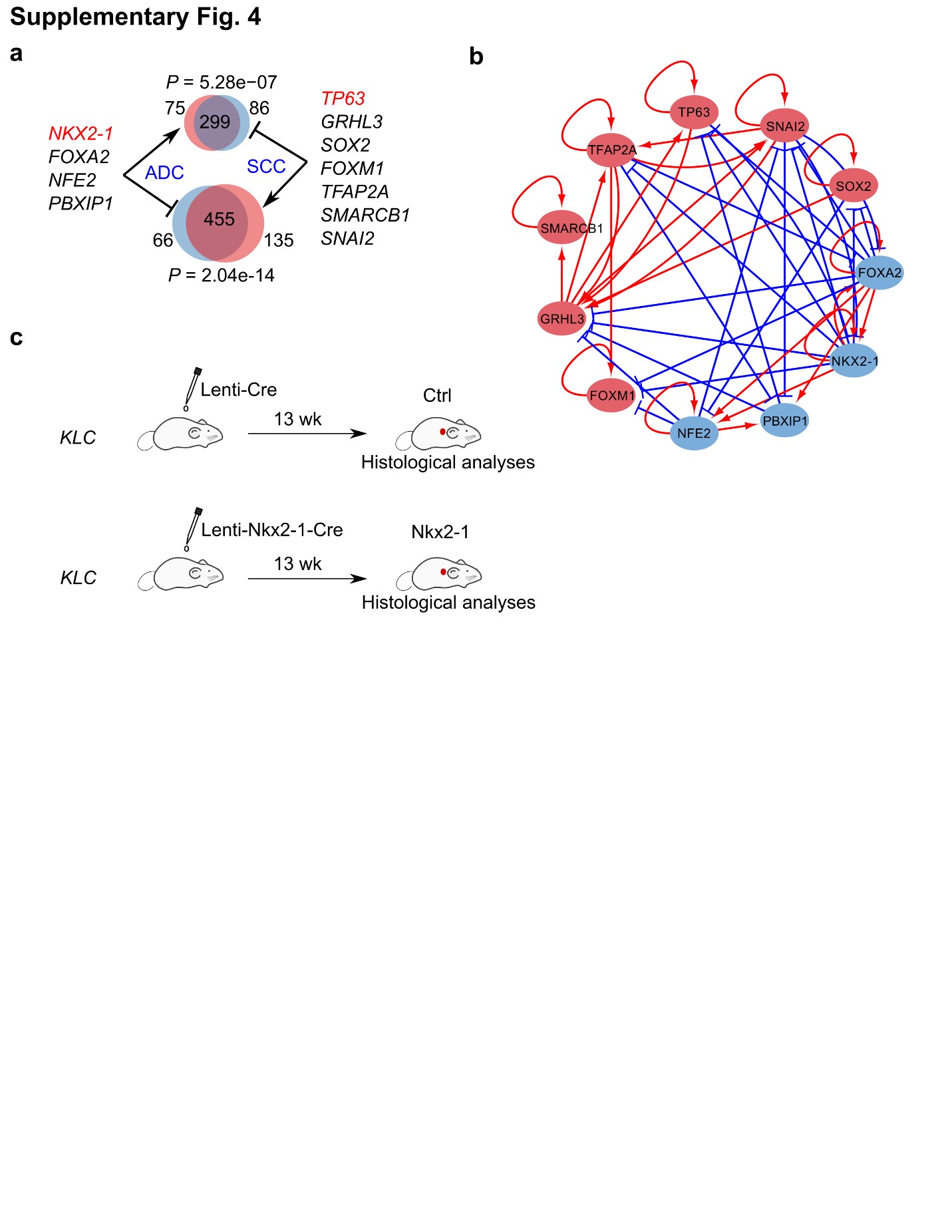


**Supplementary Fig 4. A mechanistic model of AST modulated by Wnt/β-catenin.**

**a**. Mutual repression of ADC- and SCC- specific TFs in human (see Methods). Enrichment of target genes were evaluated with Fisher’s exact test.

**b**. Regulatory network for ADC- and SCC- specific TFs. Each edge represents a direct transcriptional action.

**c**. A scheme of Lenti-Nkx2-1 transfection in *KLC* mice. Tumors were analyzed after 13 weeks of Ad-Cre treatment.


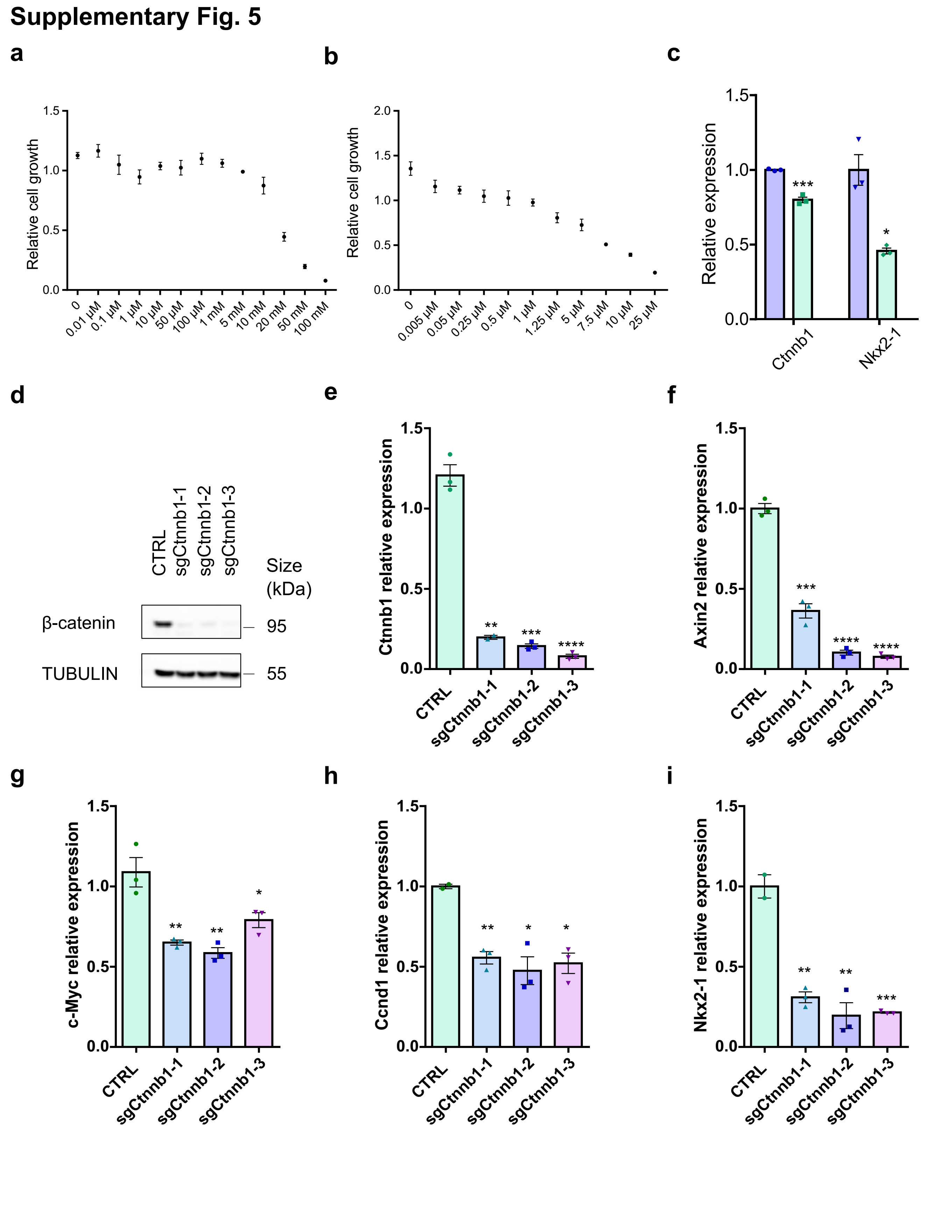
 **Supplementary Fig 5. Regulation of *Nkx2-1* expression by *Ctnnb1* knockout.**

**a**. Relative cell growth in mouse KL cell line after LiCl treatments under indicated conditions for 72 hours.

**b**. Relative cell growth in mouse KL cell line after ICG-001 treatment under indicated conditions for 72 hours.

**c.** Real-time PCR quantification of *Ctnnb1* and *Nkx2-1* gene expression in KL cells treated with ICG-001 (1.5 μM) for 48 hours. **P <* 0.05, ****P <*0.001.

**d**. Western blotting analyses of β-catenin levels in mouse KL cell line with or without sg*Ctnnb1*-1, *Ctnnb1*-2 and *Ctnnb1*-3. TUBULIN serves as the control.

**e.** Real-time PCR quantification of *Ctnnb1* gene expression in mouse KL cell line with or without sg*Ctnnb1*-1, *Ctnnb1*-2 and *Ctnnb1*-3. Data are shown as mean ± SEM. ***P <* 0.01, ****P <* 0.001, *****P <* 0.0001.

**f.** Real-time PCR quantification of *Axin2* gene expression in mouse KL cell line with or without sg*Ctnnb1*-1, *Ctnnb1*-2 and *Ctnnb1*-3. Data are shown as mean ± SEM. ****P <* 0.001, *****P <* 0.0001.

**g**. Real-time PCR quantification of *c-Myc* gene expression in mouse KL cell line with or without sg*Ctnnb1*-1, *Ctnnb1*-2 and *Ctnnb1*-3. Data are shown as mean ± SEM. **P <* 0.05, ***P <* 0.01.

**h.** Real-time PCR quantification of *Ccnd1* gene expression in mouse KL cell line with or without sg*Ctnnb1*-1, *Ctnnb1*-2 and *Ctnnb1*-3. Data are shown as mean ± SEM. **P <* 0.05, ***P <* 0.01.

**i**. Real-time PCR quantification of *Nkx2-1* gene expression in mouse KL cell line with or without sg*Ctnnb1*-1, *Ctnnb1*-2 and *Ctnnb1*-3. Data are shown as mean ± SEM. ***P <* 0.01, ****P <* 0.001.
