# Supplementary figures and images for: Oxidative stress-triggered Wnt signaling perturbation characterizes the tipping point of lung adeno-to-squamous transdifferentiation
Source: Signal Transduct Target Ther. 2023 Jan 11;8:16. doi: 10.1038/s41392-022-01227-0 (PMC9832009; doi:10.1038/s41392-022-01227-0)

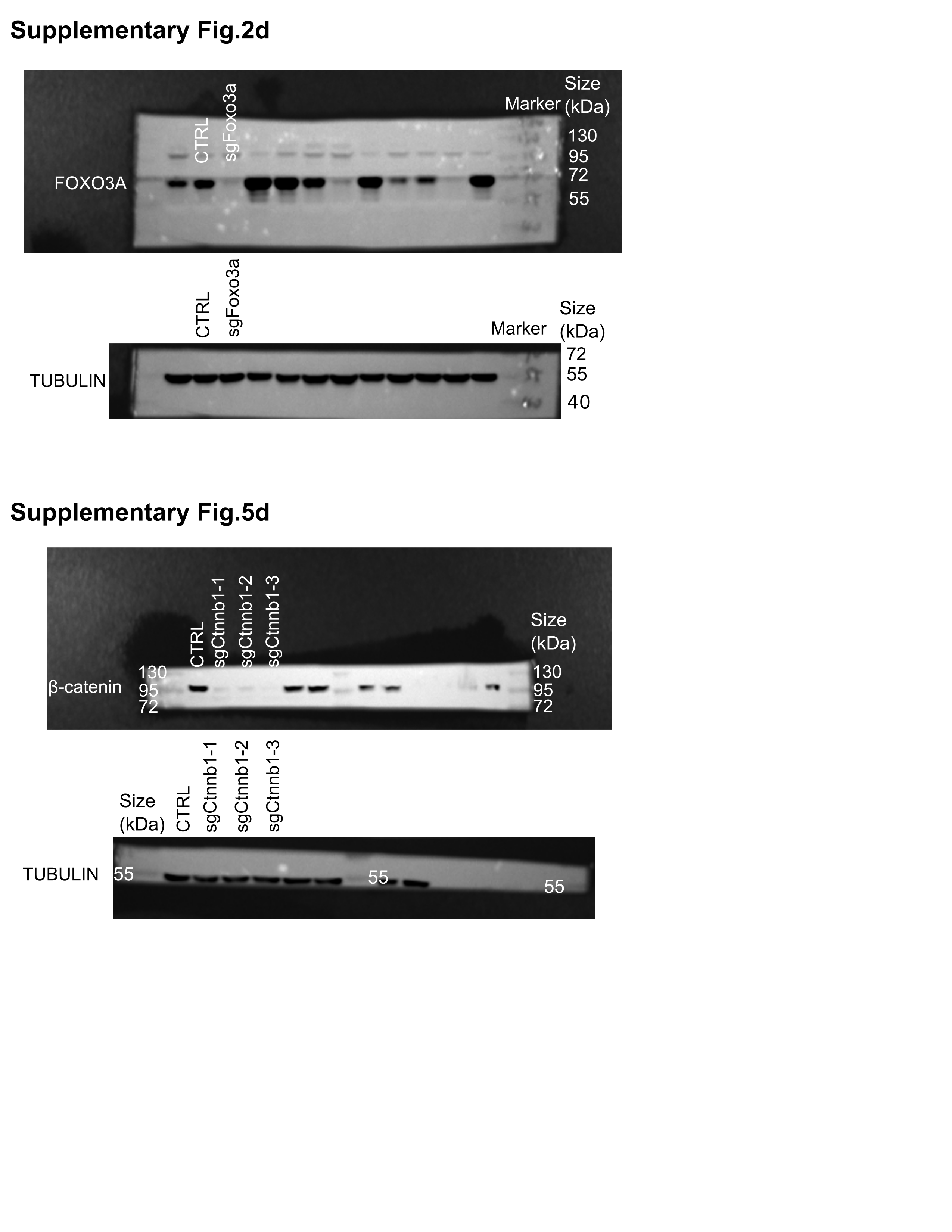

Supplement: Supplementary file 2 — Additional raw data: unprocessed immuneblot data [file 41392_2022_1227_MOESM2_ESM.tif]
